# Genome-Wide Association Study and RNA-Seq Analysis Uncover Candidate Genes Controlling Growth Traits in Red Tilapia (*Oreochromis* spp.) Under Hyperosmotic Stress

**DOI:** 10.3390/ijms26136492

**Published:** 2025-07-05

**Authors:** Bingjie Jiang, Yifan Tao, Wenjing Tao, Siqi Lu, Mohamed Fekri Badran, Moustafa Hassan Lotfy Saleh, Rahma Halim Mahmoud Aboueleila, Pao Xu, Jun Qiang, Kai Liu

**Affiliations:** 1 Key Laboratory of Freshwater Fisheries and Germplasm Resources Utilization, Ministry of Agriculture and Rural Affairs, Freshwater Fisheries Research Center of Chinese Academy of Fishery Sciences, Wuxi 214082, China; 2 Integrative Science Center of Germplasm Creation in Western China (CHONGQING) Science City, Key Laboratory of Freshwater Fish Reproduction and Development (Ministry of Education), School of Life Sciences, Southwest University, Chongqing 400715, China; 3 Aquatic Hatchery Production Department, Fish Farming and Technology Institute, Suez Canal University, Ismailia 41522, Egypt; 4 Wuxi Fisheries College, Nanjing Agricultural University, Wuxi 210095, China

**Keywords:** red tilapia, growth traits, RNA-seq, GWAS, candidate gene

## Abstract

Growth traits are the most important economic traits in red tilapia (*Oreochromis* spp.) production, and are the main targets for its genetic improvement. Increasing salinity levels in the environment are affecting the growth, development, and molecular processes of aquatic animals. Red tilapia tolerates saline water to some degree. However, few credible genetic markers or potential genes are available for choosing fast-growth traits in salt-tolerant red tilapia. This work used genome-wide association study (GWAS) and RNA-sequencing (RNA-seq) to discover genes related to four growth traits in red tilapia cultured in saline water. Through genotyping, it was determined that 22 chromosomes have 12,776,921 high-quality single-nucleotide polymorphisms (SNPs). One significant SNP and eight suggestive SNPs were obtained, explaining 0.0019% to 0.3873% of phenotypic variance. A significant SNP peak associated with red tilapia growth traits was located on chr7 (chr7-47464467), and *plxnb2* was identified as the candidate gene in this region. A total of 501 differentially expressed genes (DEGs) were found in the muscle of fast-growing individuals compared to those of slow-growing ones, according to a transcriptome analysis. Combining the findings of the GWAS and RNA-seq analysis, 11 candidate genes were identified, namely *galnt9*, *esrrg*, *map7*, *mtfr2*, *kcnj8*, *fhit*, *dnm1*, *cald1*, *plxnb2*, *nuak1*, and *bpgm*. These genes were involved in ‘other types of O-glycan biosynthesis’, ‘glycine, serine and threonine metabolism’, ‘glycolysis/gluconeogenesis’, ‘mucin-type O-glycan biosynthesis’ and ‘purine metabolism signaling’ pathways. We have developed molecular markers to genetically breed red tilapia that grow quickly in salty water. Our study lays the foundation for the future marker-assisted selection of growth traits in salt-tolerant red tilapia.

## 1. Introduction

Red tilapia (*Oreochromis* spp.) is a high-quality hybrid species with high adaptability, fast growth, and delicious meat [1]. Red tilapia is more adaptable to high-salinity conditions, except for blackchin tilapia (*Sarotherodon melanotheron*) [2]. Because red tilapia is able to tolerate a broad salinity range, it is an essential species for brackish culture, which has become a model species for investigating salinity adaptation in aquatic species [3]. In China, the lack of freshwater has limited the industry of the red tilapia in some ways. However, China is relatively rich in brackish water resources, and making appropriate use of these resources to culture salt-tolerant fish species has become an important direction for aquaculture development. Furthermore, the flesh quality of red tilapia cultured in brackish water is superior to that of those cultured in freshwater, and it has higher economic value [4]. The growth rate of Nile tilapia (*O.niloticus*) raised in saline water is significantly lower than that of those cultured in freshwater [5]. Our previous studies revealed that red tilapia show significantly reduced growth rates at salinity levels above 16% [6]. Previous studies have developed growth markers and fine-mapped growth quantitative trait loci in tilapia [7,8]. However, most earlier research focused on Nile tilapia. Less research has been conducted on important traits in red tilapia, especially its growth traits under hyperosmotic stress.

Growth traits are particularly crucial economic characteristics in fish cultivation [9]. Improving growth features can lower aquaculture’s economic costs while raising yield [10]. Therefore, improving the growth rate is an important objective of many fish-breeding studies [11]. From a genetic perspective, growth is a quantitative characteristic that is impacted by both genetic and environmental variables, controlled by numerous genetic loci, and governed by intricate physiological processes [12]. Li et al. [10] reported that there was a strong genetic basis for the growth trait, which could be improved by phenotypic selection, while selective breeding can accelerate increases in growth. Selective breeding has great potential to improve fish growth characteristics and increase economic efficiency in fisheries by accumulating genetic modifications in cultivated species [13]. Selection based on growth traits has been shown to promote the growth rate and improve the flesh color of fish, thereby increasing product quality and reducing farming costs [14]. For instance, previous studies found that selective breeding can significantly increase the body mass of rainbow trout (*Oncorhynchus mykiss*), thereby contributing to the growth of the trout industry [15]. Therefore, targeting growth traits could be significant for breeding fast-growing cultured fish varieties.

In recent years, the selective breeding of fish has changed dramatically with the development of genomic technologies. This is mainly because increasing numbers of gene loci are being discovered with the development of sequencing technology, as well as markers linked to such loci, such as single-nucleotide polymorphisms (SNPs) [16]. SNPs are abundant in the genome, and have become the most effective molecular markers in genetic linkage analysis [16]. Genome-wide association study (GWAS) is an excellent method to screen relevant SNPs linked with particular traits [17]. GWAS has been extensively applied to study genetic variation in economically critical traits, such as disease resistance, stress tolerance and growth [18,19,20]. Researchers have used GWAS to study growth traits in several fish species, such as mandarin fish (*Siniperca chuatsi*), olive flounder (*Paralichthys olivaceus*), sea bass (*Dicentrarchus labrax*) and yellow catfish (*Pelteobagrus fulvidraco*) [21,22,23,24]. Transcriptomic is a key tool for studying the relationship between gene expression and target phenotypes. GWAS combined with transcriptome sequencing can effectively narrow down target regions to specific quantitative trait locus (QTL) intervals, thus improving the accuracy of their localization [25]. It was widely utilized in farmed fish, like tapertail anchovy (*Coilia nasus*) [26], snakehead (*Channa argus*) [27] and *O. mossambicus* × *O. niloticus* [28]. However, this technique has not yet been employed to investigate the genetic basis of growth characteristics in red tilapia.

In our research, we used the combination of GWAS and transcriptome assay to characterize and validate candidate genes related to growth traits in red tilapia under hyperosmotic stress. Firstly, 160 red tilapia were genotyped for GWAS to discover several growth-related SNPs and candidate genes. Subsequently, a comparative transcriptome analysis of male and female fish with extremely large (fast-growing group) and extremely small body sizes (slow-growing group) was conducted to characterize differentially expressed genes (DEGs), as well as to determine the candidate genes involved in the growth of red tilapia. Finally, the results from both analytical methods were combined to identify overlapping critical candidate genes involved in growth. These SNPs and potential candidate genes offer fresh perspectives on the growth mechanism of salt-tolerant red tilapia, along with effective genetic markers. This study offers a theoretical basis for culturing red tilapia strains with faster growth in saline water.

## 2. Results

### 2.1. Statistics of Growth Traits

The descriptive data for the four growth traits are shown in Table S1. The average values of total body length, body height, body mass and caudal peduncle height were 238.99 ± 1.64 mm, 78.52 ± 9.97 mm, 305.16 ± 6.69 g and 29.00 ± 0.25 mm, respectively. All four growth trait pairs had Pearson’s correlation coefficients between them ranging from 0.616 to 0.897 (Table 1), and the positive pairwise correlations were statistically significant (*p* < 0.001). The phenotypic values of all four growth traits were approximately normally distributed in the population (Figure S1A–D), indicating that the samples could be used for the GWAS. We analyzed the dispersion of four growth traits between females and males, to determine if red tilapia exhibit sexual dimorphism. As shown in Figure S2A–C, the total body length, body height, and body mass were significantly different (*P* ≤ 0.05) between male and female fish, and the median of these traits was higher in males than in females (Figure S2A–D). These findings indicated a potential link between biological sex and growth in red tilapia.

**Figure 1:**
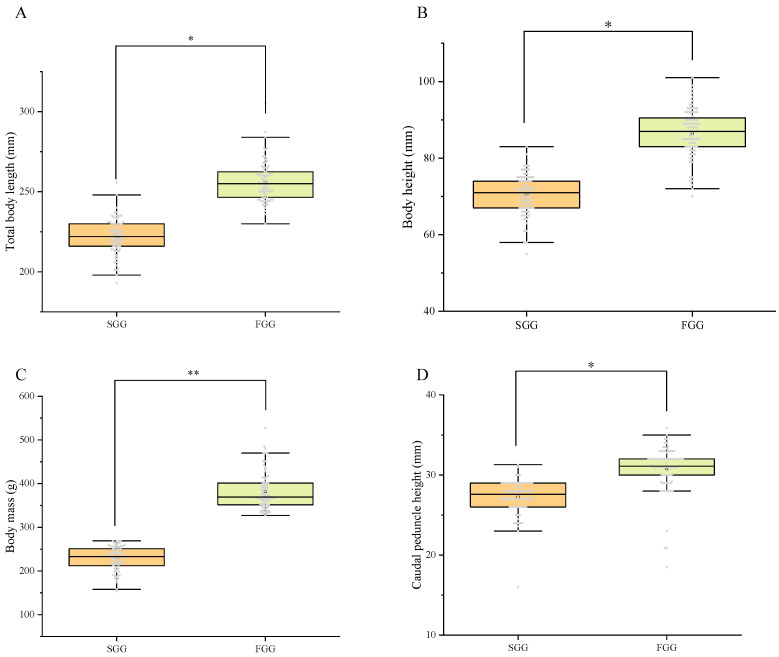
The comparison of growth traits in red tilapia between the fast-growing and slow-growing groups. Note: FGG: fast-growing group; SGG: slow-growing group; **: *p* < 0.001; *: *p* < 0.05; Each grey dot represented an individual.

Two groups of red tilapia with significant growth differences were selected from the same breeding population, i.e., 80 fast-growing individuals with body mass ranging from 327 g to 528 g, and 80 slow-growing individuals with body mass ranging from 153 g to 269 g. Figure 1 displayed the four growth traits of each individual in detail. The average values of growth traits in the fast-growing individuals were as follows: total body length, 256.13 ± 1.39 mm; body height, 86.64 ± 0.70 mm; body mass, 381.77 ± 4.71 g; and caudal peduncle height, 30.82 ± 0.33 mm. The average values of growth traits in the slow-growing individuals were as follows: total body length, 221.85 ± 1.23 mm; body height, 70.41 ± 0.58 mm; body mass, 228.60 ± 3.06 g; and caudal peduncle height, 27.18 ± 0.24 mm (Figure 1). Among these four growth traits, the difference between the fast-growing and slow-growing groups was significantly different (*p* < 0.05).

### 2.2. Quality Control for the Resequencing Data and Genotyping

**Figure 2:**
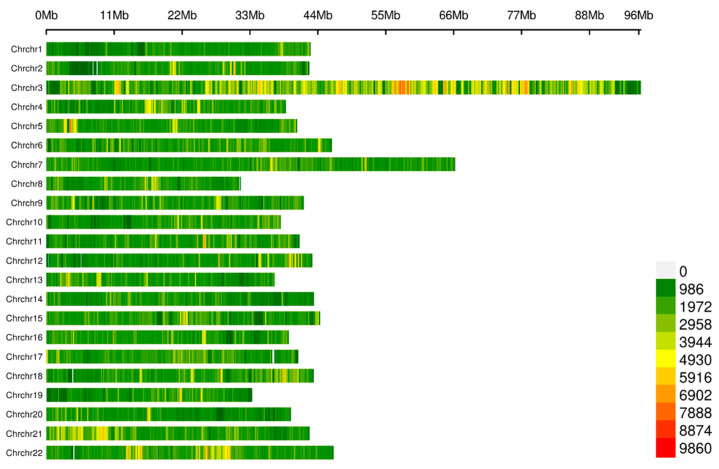
The density and distribution of the high-quality SNPs genotyped on each chromosome of red tilapia. Notes: red denotes high density, yellow denotes moderate density, and green denotes low density.

The whole genomes of 160 fish were sequenced, yielding 130.36 billion raw reads, with an average of 0.81 billion raw reads per individual (Supplementary File S1). After filtering, a total of 123.05 billion clean reads were obtained. The average GC content was 41.04%, and the average Q20 and Q30 values were 98.96% and 96.63%, respectively. Subsequently, a total of 17,963,708 SNPs were identified and 12,776,921 SNPs were retained after quality control (Supplementary File S2). The red tilapia’s 22 chromosomes (chr) had an equal distribution of SNPs (Figure 2). The highest number of SNPs (2,768,822) was on chr 3, while the lowest number (509,284) was on chr 19. The presence of these high-density SNPs provided an important foundation for the development of molecular markers associated with fast growth under hyperosmotic stress.

### 2.3. Analysis of Population Structure

**Figure 3:**
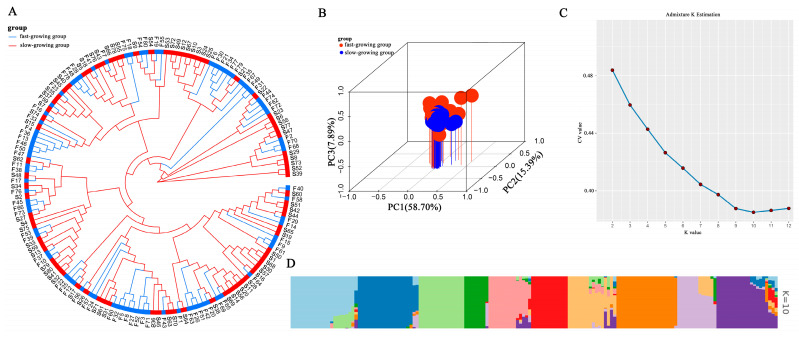
Population structure analysis. (**A**): The phylogenetic tree analysis of genotype in the red tilapia. (**B**): The PCA scatter plot revealed the spread of red tilapia populations; each point represented a sample, with red and blue points indicating the slow-growing and fast-growing groups, respectively. (**C**): The K-value for CV error estimation ranges from 2 to 12. (**D**): The population genetic structure analysis of the red tilapia (K = 10). Each column indicated an individual; The lengths of the different colored segments indicated the proportion of that individual’s genome that is attributable to a particular ancestor.

High genetic diversity in red tilapia was indicated by the LD decay study, which showed a sharp drop in LD coefficients with a 50% fall at a distance of 376.9 kb (Figure S3A). The genetic background and genetic correlations of red tilapia were analyzed by principal component analysis (PCA), kinship analysis, and population structure analysis. According to the first three PCAs, there was no discernible population stratification in the red tilapia population, indicating that its members had a similar genetic background (Figure 3B). The phylogenetic tree based on the NJ method indicated no obvious branching aggregation in the red tilapia population (Figure 3A). The cross-validation error value of K showed a decreasing trend during the genetic structure analysis, reaching a minimum at K = 10 (Figure 3C,D). In addition, the red tilapia population exhibited weak genetic correlations, with the majority of the values ranging between 0.0 and 0.25 (Figure S3B). Overall, the genetic background of the red tilapia population resembled that of a natural population, and there was no significant artificial disturbance or selection.

### 2.4. GWAS for Growth Traits

**Figure 4:**
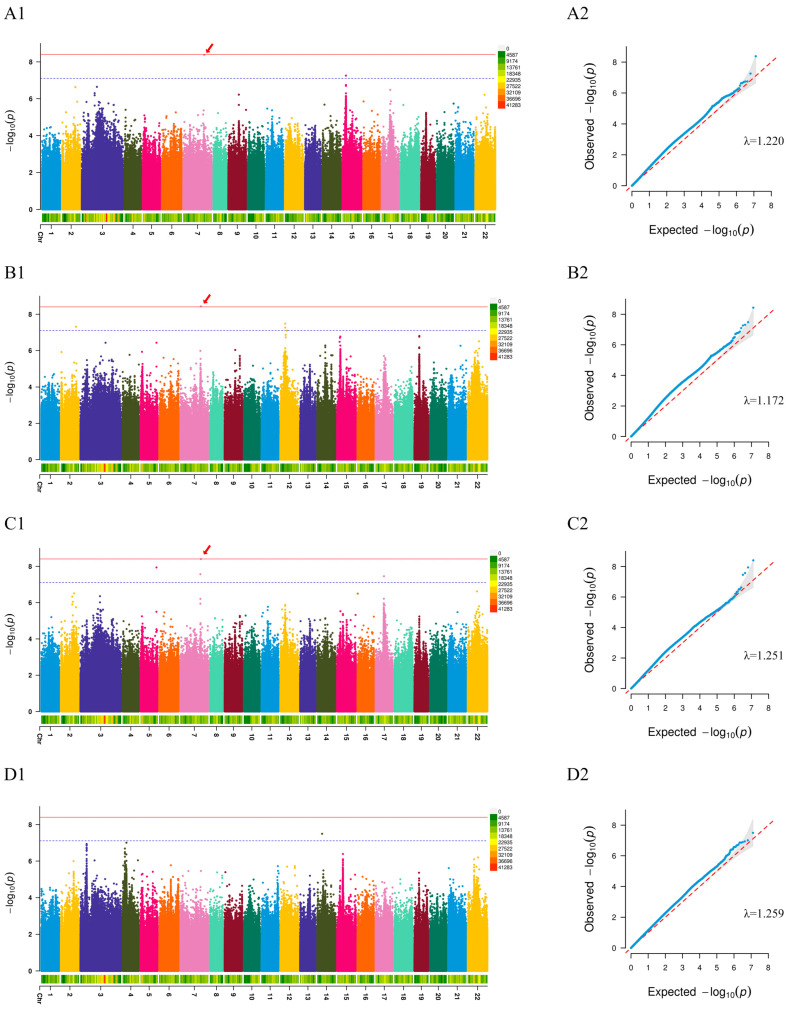
Manhattan plot (**A1**–**D1**) and QQ plot (**A2**–**D2**) for GWAS, using the total body length (**A**), body height (**B**), body mass (**C**), and caudal peduncle height (**D**) of red tilapia. The red solid line indicated the significance threshold line; the blue dashed line denoted the suggestive threshold line.

A GWAS was conducted for each of the four growth traits using the Farmcpu model, combining the first three principal components (PCs) and the biological sex as covariates. The inflation factor (λ) values for the four growth-related traits, i.e., total body length, body height, body mass, and caudal peduncle height, were 1.220, 1.172, 1.251, and 1.259, respectively (Figure 4A2–D2). These results approximated the expected *p*-value expansion coefficient of 1. The calculated threshold for a significant association between an SNP and a growth trait was −log10 (0.05/12,776,921) = 8.41, and the threshold for a suggestive association was −log10 (1/12,776,921) = 7.11. As shown in the Manhattan plots, nine SNPs achieved the suggestive threshold related to the four growth characteristics (Figure 4A1–D1). Among them, chr 7 and chr 15 had two significant SNPs linked to total body length. Four significant SNPs were associated with body height, including two SNPs on chr 2 and chr 7, and two SNPs on chr 12. There were four significant SNPs on chr 5, chr 7, and chr 17 that were linked to body mass. For caudal peduncle height, one significantly associated SNP was located in chr 14. Interestingly, chr7-48631309 was related to three growth traits (total body length, body height and body mass), and was located on the intergenic region of the *patatin-like phospholipase domain containing 8* (*pnpla8*) gene (Table 2). Additionally, three SNPs were found in the intronic regions of three genes; *neuroblastoma-amplified sequence* (*nbas*), *plexin-b2* (*plxnb2*), and *limbic system-associated membrane protein* (*lsamp*). The rest of the five SNPs were positioned in the intergenic region. One SNP was found in the upstream region of the *acyl-coenzyme A thioesterase 1* (*acot1*) gene.

### 2.5. Fine Mapping of Candidate Regions

**Figure 5:**
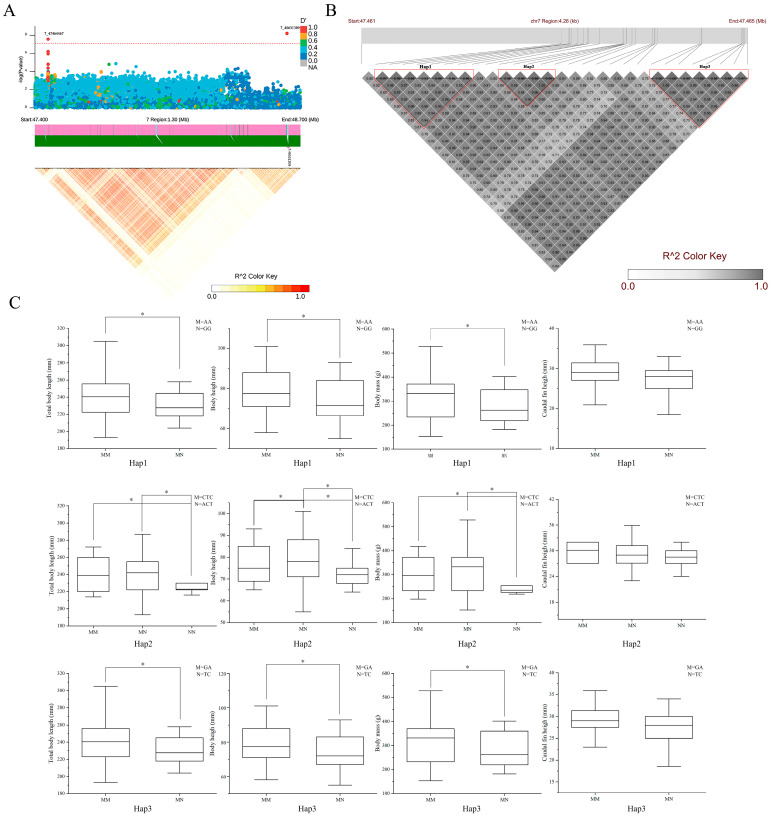
The fine localization of the candidate region of chr 7. (**A**) The locus zoom plot revealed fine localization near significant SNPs in chr 7. Red dots denoted SNP signals above the significance threshold, other color dots were below the threshold. (**B**) Haplotype block counting of QTL areas with significant SNP spikes on chr7. The black depth of the square increased with the value of r2. (**C**) The distribution of growth traits of different haplotypes; *: *p* < 0.05.

To further narrow down the candidate interval to pinpoint key genes, LD analysis was performed on SNPs near the lead SNP (chr7-47464467). The results revealed that the locally formed haplotype regions were enriched with these SNPs, which were highly interlocked with each other and showed a high degree of disequilibrium (Figure 5A). In addition, the SNPs with strong LD relationships with the lead SNP (chr7-47464467) were all located near *plxnb2* (D′ > 0.95). The SNP markers in coding sequence regions and SNPs substantially linked to four growth traits were extracted for haplotype analysis (Figure 5B, Supplementary File S3). A total of three haplotypes were obtained, namely Hap.1, Hap.2, and Hap.3. For Hap.1, individuals of the AA/AA heterozygous genotype showed significantly greater growth traits than individuals of the AA/GG heterozygous genotype (Figure 5C). For Hap.2, the growth traits of individuals with the ACT/ACT heterozygous genotype were considerably smaller than those of individuals with the CTC/ACT heterozygous and CTC/CTC homozygous genotypes (Figure 5C). For Hap.3, individuals of the GA/GA genotype had significantly higher growth traits compared to those with the GA/TC heterozygous genotype (Figure 5C). Therefore, *plxnb2* was recognized as a key candidate gene significantly related to growth.

### 2.6. Transcriptome Analysis

Considering that the growth traits differed between males and females, a sex-based transcriptome sequencing approach was employed to reduce the influence of biological gender on the outcome. Three FM, three FF, three SM and three SF were selected for transcriptome sequencing. As shown in Table S2, the fast-growing groups exhibited significantly larger average total body length, body height, body mass and caudal peduncle height compared to the slow-growing groups (*p* < 0.05). Subsequently, a mean of 50,954,275, 61,552,916, 61,997,055, and 56,024,974 clean reads were acquired from the FM, FF, SM, and SF libraries, respectively. After strict quality control, the proportion of Q30 (error rate < 0.1%) was greater than 96.16%, and more than 94.98% of the clean reads were able to be aligned with the reference genome (Supplementary File S4).

**Figure 6:**
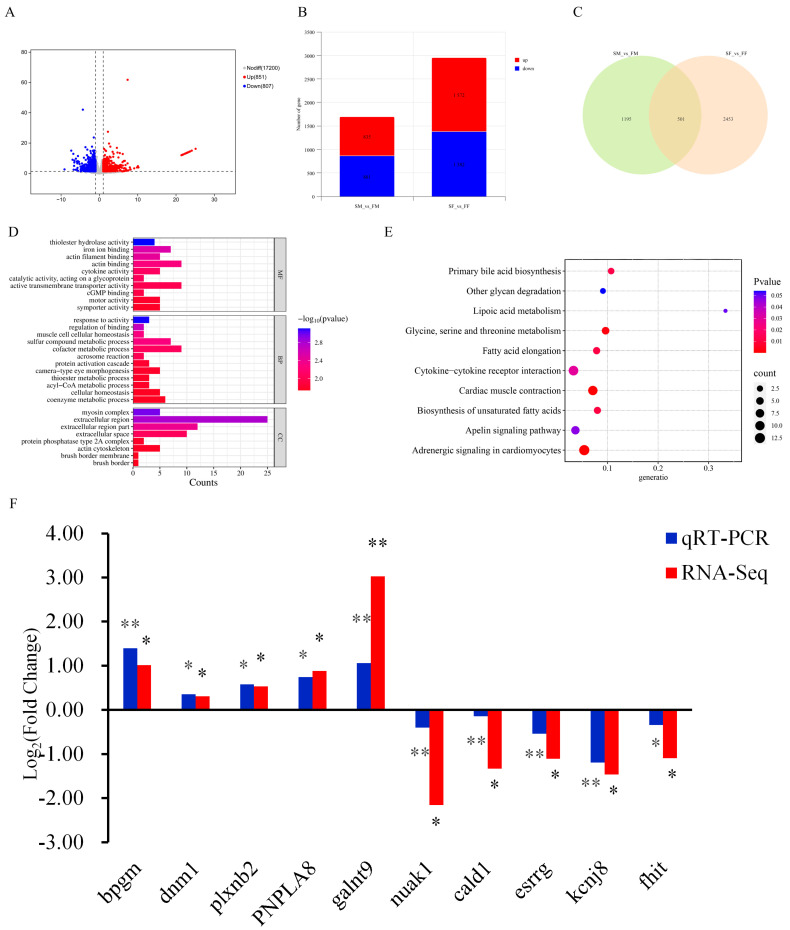
Transcriptome analyses based on fast-growing and slow-growing groups of red tilapia. (**A**) Volcano plot of DEGs in the fast-growing and slow-growing groups for muscle in red tilapia. (**B**) Histogram of DEGs in the female (SF vs. FF) and male (SM vs. FM) comparison group for muscle in red tilapia. (**C**) Venn maps of DEGs in the female (SF vs. FF) and male (SM vs. FM) comparison group for muscle in red tilapia. (**D**) The top 30 GO terms of 501 overlapping DEGs for muscle in red tilapia. (**E**) The top 10 KEGG pathways of 501 overlapping DEGs for muscle in red tilapia. (**F**) The qRT-PCR validation for 10 DEGs in red tilapia; **: *p* < 0.001; *: *p* < 0.05;.

A total of 1656 DEGs were identified between the fast-growing and slow-growing groups, comprising 807 down-regulated and 851 up-regulated DEGs (Figure 6A). Next, we identified DEGs between the slow-growing and fast-growing males (3 SM vs. 3 FM) and between the slow-growing and fast-growing females (3 SF vs. 3 FF). In these comparisons, there were 2,954 DEGs (1,572 up-regulated, 1,382 down-regulated) identified in females and 1,696 DEGs (835 up-regulated and 861 down-regulated) found in males (Figure 6B). In addition, 501 DEGs were shared between these two groups, and were identified as those that are likely related to the growth of red tilapia (Figure 6C). The 501 DEGs were enriched in 143 critical GO terms (*p* < 0.05, Supplementary File S5). Figure 6D displayed the top 30 enriched GO terms. The most enriched GO terms were ‘myosin complex’, ‘muscle cell cellular homeostasis’, ‘actin filament binding’, ‘actin binding’, and ‘actin cytoskeleton’ (Figure 6D). In the KEGG enrichment analysis, eight pathways had a substantial DEG enrichment (*p* < 0.05, Supplementary File S6). The enriched pathways included ‘fatty acid elongation’, ‘fatty acid metabolism’, and ‘fatty acid biosynthesis’, all of which are involved in lipid metabolism. These findings implied that lipid metabolism plays a significant role in the growth of red tilapia (Figure 6E).

Based on the RNA-seq results, 10 DEGs with substantial differences in transcript levels in muscle tissue between the slow- and fast-growing male groups were chosen for qRT-PCR confirmation (Figure 6F). The RNA-seq and qRT-PCR results were log2 normalized and correlated in SM vs. FM groups, and the data from the two analyses showed a highly significant correlation (*p* < 0.05). The expression trends of the 10 genes observed in the qRT-PCR results were consistent with those detected in the RNA-seq analysis, confirming the reliability of the sequencing results.

### 2.7. Integration Analysis of GWAS and Transcriptome

**Figure 7:**
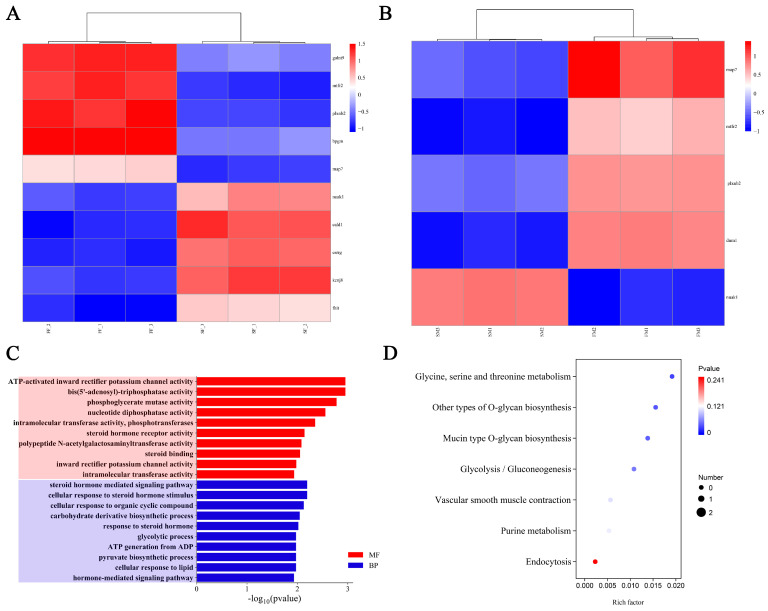
Eleven candidate genes for combined GWAS and RNA-seq analyses. (**A**) Hierarchical cluster analysis of the candidate genes in SF vs. FF comparison group. (**B**) Hierarchical cluster analysis of the candidate genes in SM vs. FM comparison group. (**C**) The top 20 GO terms of the 11 candidate genes. (**D**) The KEGG pathways enriched with 11 candidate genes.

The 100 kb region upstream and downstream of significant SNPs was chosen as the candidate interval for genes associated with growth. In these areas, 182 potential genes were identified and annotated. These genes were compared with the DEGs detected in any of the comparisons (fast-growing vs. slow-growing, SF vs. FF and SM vs. FM), and those that overlapped were selected as the final candidate genes. In total, we identified 11 final candidate genes (Figure 7A,B). Among them, *dnm1* was significantly expressed only in male individuals (SM vs. FM), whereas *map7*, *mtfr2*, *nuak1*, and *plxnb2* were expressed in both female and male individuals (SF vs. FF, SM vs. FM). The discovery of these genes could be helpful for conducting more thorough studies on the molecular mechanisms of growth traits in red tilapia populations in saline water. These candidate genes were subjected to GO and KEGG enrichment analyses (Figure 7C,D). The findings indicated that a number of GO terms might be associated with the growth of red tilapia, including ‘cellular response to lipid’, ‘ATP-activated inward rectifier potassium channel activity’, ‘ATP generation from ADP’, ‘glycolytic process’, and ‘steroid hormone-mediated signaling pathway’ (Figure 7C). Additionally, the candidate genes were enriched in seven KEGG pathways, including five that may be relevant to growth: ‘glycine, serine and threonine metabolism’, ‘other types of O-glycan biosynthesis’, ‘glycolysis/gluconeogenesis’ ‘mucin-type O-glycan biosynthesis’ and ‘purine metabolism’ (Figure 7D).

## 3. Discussion

As a suitable culture species for saline waterin China, red tilapia has high economic value. In our study, it was found that red tilapia exhibited significant growth differences among individuals, especially in the total body length, body height, body mass and caudal peduncle height (*p* < 0.05). Additionally, tilapia exhibited sexual dimorphism in growth capacity, with male tilapia showing significantly faster growth rates than females [29]. In this study, the male red tilapia had much greater growth traits than females, including the total body length, body height and body mass (*p* < 0.05). These are similar to the growth traits of other tilapia, like Nile tilapia [30] and blue tilapia [31]. Therefore, it was established that the sexuality of red tilapia influences its growth. To avoid the effect caused by sexuality differences, sexuality was used as a covariate during GWAS analysis in our study, and the transcriptome was divided into male and female groups for differential expression analysis.

The structure of the population might lead to positive association analyses, resulting in non-chained associations between these markers [32]. Therefore, population structure should be considered in conjunction with the kinship matrix to improve the correlation between SNPs and phenotypes when performing genetic variation. This approach has been extensively investigated in *Larimichthys crocea*, such as the identification of genes associated with visceral white spots and the study of hypoxia tolerance traits [33,34]. Therefore, the present study investigated the genetic relationships and population structure of red tilapia. Subsequently, in order to reduce false positives, a MLM was used for GWAS analysis of the red tilapia population [35]. Our results revealed that the genome inflation factor approached 1, remarkably, certainly proving the complete calibration of the population stratification and further proving the stability and accuracy of the model. On this basis, the SNPs with LD values were screened using high markers. Here, we identified 7.11 as the genome-wide threshold of suggestive.

As we all know, the growth of fish was controlled by more than one gene [36]. Similar results have been reported in previous studies. For example, 23 and 26 growth-associated SNPs were detected in *Epinephelus fuscoguttatus* and *E. lanceolatus*, respectively, and distributed in multiple chromosomes [12,37]. In *Takifugu bimaculatus*, 18 QTL linked to growth traits were identified on LG7, LG10 and LG21 [38]. We also identified a total of nine growth-associated SNPs on multiple chromosomes in red tilapia, including one significant and eight suggestive SNPs in the total body length, body height, body mass and caudal peduncle height. Similarly to other bony fishes, growth traits in red tilapia were controlled by multiple genes. In contrast, there were large differences in the individual development of red tilapia, which could be attributed to a variety of mutations. In particular, the effect sizes of these SNPs were comparatively low, with the phenotypic variation values varying from 0.0019% to 1.3369%, which were lower than other fishes such as *E. lanceolatus* (7.09–18.42%), *Seriola lalandi* (2.6–4.9%), and *E. coioides* (14.5–29.1%), *etc.* [37,39,40]. It was possible that the growth-related phenotype in red tilapia was regulated by many small effector genes and complex molecular mechanisms. These SNPs were expected to provide a theoretical basis for enriching the growth dominance variation in red tilapia, and would provide valuable molecular markers for its molecular breeding.

Notably, the GWAS identified a number of candidate genes associated with growth, such as *pnpla8*, *elf3*, and *acot1*. The Pnpla8 is a member of the phospholipase family that catalyzes the cleavage of membrane phospholipids by FA. It can preferentially act on arachidonic acid-containing membrane phospholipids to hydrolyze and cleave them to free fatty acids and lysophospholipids [41]. Thus, the *pnpla8* plays an essential role in mobilizing arachidonic acid and releasing lipid second messengers in response to cellular stimuli [42]. In rabbit proximal tubule cells, the inhibition of the *pnpla8* gene expression resulted in lipid peroxidation and cellular turnover [43]. In addition, the overexpression of the *pnpla8* gene was revealed to significantly reduce hepatic steatosis by increasing autophagy, and the SREBP-2/PNPLA8 axis represented a novel mechanism for the regulation of lipid homeostasis. The *elf3* gene impeded lipogenic differentiation by regulating the PI3K/AKT pathway [25]. The higher *elf3* mRNA abundance at 230 DIM goats might be related to the role of lipids in increasing mammary cell apoptosis [7]. Similar results have been observed in human breast cancer [44]. The Acot1 is a cytoplasmic thioesterase that converts acyl coenzyme A to fatty acids and CoA, and is a central node in the interactome of the transition from steatosis to MASH (metabolic dysfunction-associated steatohepatitis) [45]. The expression of acot1 is 3-fold higher in MASH patients than in steatosis [46]. In mice, the upregulation of the *acot1* gene led to MASH by increasing glycerophospholipid accumulation, while the downregulation of the *acot1* gene prevented MASH development [46]. In addition, the *plxnb2* gene was identified near the lead SNPs (chr7-47464467) on chr 7, which might also be associated with growth and development of red tilapia. The Plxnb2 is a transmembrane protein found in various tissues [47]. Recent studies have demonstrated that the *plxnb2* gene was abundantly present in the growth plate of rats, as well as possibly being involved in their chondrogenesis, skeletogenesis, and bone remodeling [48]. Therefore, these genomic regions could be candidate functional areas for the regulation of various growth traits in red tilapia.

With the development of histological technologies, single-omics studies are potentially biased and have limitations in revealing the intrinsic mechanisms of fish growth and development. Multi-omics approaches have been used to uncover important molecular markers for target traits and to discover potential candidate genes. A total of 11 candidate genes were found to be associated with four growth traits by transcriptome and GWAS analysis. These candidate genes were significantly enriched in the KEGG signaling pathways, including Glycine, serine, and threonine metabolism, other types of O-glycan biosynthesis, mucin type O-glycan biosynthesis, glycolysis/gluconeogenesis, and purine metabolism. The glycolysis/gluconeogenesis is a central metabolic pathway that generates energy for cellular activities [49]. The expression level of the glycolytic enzyme (Bpgm) was significantly higher in fast-growing than in slow-growing groups. Previous studies in *Danio rerio*, *P. stellatus*, and *Sus scrofa* have illuminated the effect of glycolysis in enhancing muscle growth [50,51,52]. In addition, the synthesis of O-glycans has a positive effect on fat deposition, with a high stability of lipase on O-pentynylglucan, resulting in an influence on fat metabolic effects [53]. Among them, the *galnt9* gene is mainly involved in the transfer of the N-acetylgalactosamine motif on the donor uridine diphosphate N-acetylgalactosamine to the acceptor protein, which binds to the Ser or Thr hydroxyl group in the specific sequences of the polypeptide chain to constitute the O-glycan. In the present study, the RNA-seq results indicated that the expression level of *galnt9* was higher in individuals of the fast-growing compared to the slow-growing group. It indicated that the *galnt9* gene might be actively involved in the synthesis of O-glycans to promote fat deposition. In addition, the high expression of the *dnm1* gene promoted cell growth in galactose carbon sources [54]. This evidence further confirms that the individuals in the fast-growing group were able to provide energy more efficiently and undergo a higher regulation of lipid deposition through glycolysis compared to the slow-growing group.

## 4. Materials and Methods

### 4.1. Fish Management

To construct a suitable local population, wild individuals were collected in Taiwan from 2010 to establish the primary parental population. This population was introduced and cultured at the Freshwater Fisheries Research Center (FFRC, Wuxi, Jiangsu, China), affiliated with the Chinese Academy of Fishery Sciences. All individuals retained distinct regional breed characteristics, and growth-selected populations were established by artificial breeding. Subsequently, mature fish were chosen as parents out of such established populations and backcrossing produced four progeny populations. After the hatching of fertilized eggs, 3000 fish fry per progeny group were taken randomly and transferred to ponds for culture. Subsequently, 300 apparently healthy fish (mean body weight: 120.72 ± 0.50 g) were randomly collected and allowed to adapt for 1 week in a concrete pond, with dimensions of 9.5 m × 4 m × 1 m (length × width × depth). Based on a pre-experiment, the final salinity of the culture water was set to 16‰ [6]. Sea crystals (Lanhaixing Co. Ltd., Hangzhou, China) were added to increase and adjust the salinity at a rate of 5‰ per day to the required salinity level [55]. The salinity level was measured daily using a salinometer (HQ4300; HACH, Loveland, CO, USA). Commercial floating feed (Zhejiang Xinxin Tianen Aquatic Feed Co., Ltd., Jiaxing, China) was provided three times daily (08:00, 12:00 and 18:00) at a rate of approximately 2–5% of fish body mass. Throughout the acclimation and culture process, the photoperiod was kept at 12 h light/12 h dark, while the dissolved oxygen content, water temperature, and pH were kept at 7.0 ± 0.5 mg/L, 26 °C ± 1 °C and 7.6 ± 0.2, respectively.

### 4.2. Growth Performance Analysis and Sampling

Four growth indexes, i.e., total body length, body height, body mass, and caudal peduncle height of the fish, were measured after 90 days of culture. The 80 fish in the fast-growing group (those with the highest growth rate) and the 80 fish in the slow-growing group (those with the slowest growth rate) were screened on the basis of body mass. Their caudal fins were gathered and stored in 100% anhydrous ethanol until DNA extraction. Subsequently, three each of the largest females and largest males, and three each of the smallest females and males, were selected and anesthetized with MS-222 (100 mg/L). Their muscle tissues were taken and stored at −80 °C until RNA extraction.

### 4.3. Library Construction and Sequencing

A standard phenol/chloroform method was used to extract total DNA from each caudal fin sample [1]. Subsequently, the sequencing libraries were constructed using TruSeq DNA PCR-Free Prep Kit reagents (Illumina, San Diego, CA, USA). Briefly, the DNA fragments were subjected to end-polishing, polyA addition, sequencing adapter ligation, purification and PCR amplification before Illumina sequencing. The library quality was assessed using the Agilent High Sensitivity DNA Kit (Agilent, Palo Alto, CA, USA) and quantified with a Quant-iT PicoGreen dsDNA Assay Kit (2 nM) (Invitrogen, Carlsbad, CA, USA). Finally, the libraries were sequenced (2 × 150 bp) using a NovaSeq sequencer (Illumina, San Diego, CA, USA).

### 4.4. Sequence Data Filtering and Genotyping Based on Single-Nucleotide Polymorphisms

Fastp v 0.20.0 software was used to generate high-quality reads, which was achieved by stripping out the sequencing adapters, unknown bases (N) ratios > 10%, all A bases, and low-quality (the number of bases with a quality value of Q ≤ 20 accounted for more than 50% of the entire reads) reads. Subsequently, these high-quality reads were compared with the Nile tilapia reference genome (NCBI number: GCF_001858045.2) using the BWA (0.7.12-r1039) software [56]. GATK v4.1.8.1 software was employed to eliminate duplicate readings in order to improve the accuracy of SNP prediction [57]. Finally, the SNP locations were annotated by ANNOVAR (version 2018Apr16, https://annovar.openbioinformatics.org/en/latest/, accessed on 9 June 2025).

### 4.5. Population Structure Analysis and Linkage Disequilibrium

To elucidate the phylogenetic relationships, the filtered SNP data were analyzed by PCAusing GCTA v1.25.3 software [58], and visualized using RStudio v4.4.1 software. The phylogenetic tree was constructed with 100 bootstraps using the maximum likelihood algorithm with FastTree v2.1 software. Admixture v1.3.0 software [59] was applied to the genetic structure of the red tilapia population, with K = 2–12 model selection. Based on the cross validation (CV) error values of different K values, the K value closest to the true value was determined. The identical-by-state (IBS) similarity between two individuals was performed by Plink v 1.9 [60], and a genetic distance (1-IBS) matrix was generated. The linkage disequilibrium (LD) and R-squared correlation coefficient (r^2^) between each pair of SNPs were calculated using PopLDdecay v 3.41 software, and an LD decay plot was generated [61].

### 4.6. Genome-Wide Association Study

The GWAS was conducted using a mixed linear model (MLMs) with GCTA software v 1.93.2 based on the inter-population SNPs and LD, and the SNPs or candidate genes that are strongly associated with growth traits were found. The formula for the MLM was as follows:

Y = Xβ + Gγ + Zu + ε

where Y is the phenotype observation vector; X represents the fixed-effects design matrix, including the first three principal components and biological sex; β is the vector of regression coefficients; G represents the covariance matrix; γ represents the corresponding coefficient vector; Z represents the design matrix for random effects; urepresents the random effects vector of relatives; and ε is the residual effect. The genome-wide significant and suggestive association thresholds were established at 0.01/N and 0.05/N, respectively, where N indicates the total number of SNPs used in the GWAS analyses. The Bonferroni correction method was used to generate these thresholds [62]. The quantile–quantile (Q-Q) plot of *p*-value and the Manhattan plot of significant SNPs were constructed using CMplot v4.3.1 software.

### 4.7. RNA Extraction and Transcriptome Sequencing Analysis

Based on the results of growth phenotyping, significant variations existed between the females and males of red tilapia. To avoid the influence of biological sex on growth traits, male and female fish were grouped separately for RNA-seq analysis. Three particularly large females (fastest growing female fish, FF) and three particularly large males (fastest growing male fish, FM), and three extremely small females (slowest growing female fish, SF) and three extremely small males (slowest growing male fish, SM) were selected. Their growth phenotypic data are shown in Table S2. Their muscle tissues were harvested and the total RNA was separated by TRIzol (Invitrogen). A UV spectrophotometer (NanoDrop One, Thermo Fisher Scientific, Waltham, MA, USA) was used to detect the concentration of RNA after its quality and purity were evaluated using 1% *w*/*v* agarose gel electrophoresis. Later, the RNA was reverse-transcribed into cDNA using a reverse transcription kit (ABM, Richmond, BC, Canada).

The twelve transcriptome libraries were sequenced using the Illumina Hiseq platform (Illumina, USA) after generating clusters. The bipartite sequencing reads were matched to the Nile tilapia reference genome using HISAT2 v2.0.1 software following quality control of the raw data. The read counts for each gene were calculated using RSEM v1.1.17 software, and then used FPKM (Fragments Per Kilo bases per Million fragments) to standardize the expression [31]. The DEGs were identified by the DESeq R software v 1.10.1 [63], and those with adjusted *p*-values < 0.05 and |log2Fold Change| > 1 as differentially expressed. The functional enrichment analyses of DEGs were conducted using the GO and Kyoto Encyclopedia of Genes and Genomes (KEGG) databases. The GO and KEGG enrichment analyses were regarded as significant when the Benjamini-Hochberg corrected *p*-value was < 0.05.

### 4.8. Validation of Candidate Genes by RT-qPCR

In order to verify the reliability of transcriptome sequencing, 10 DEGs with large differences in their transcript levels were chosen for qRT-PCR analysis. The primers were performed on Primer Premier 6.0 software (Table S3). As directed by the manufacturer, the qRT-PCR analyses were carried out using SYBR^®^ premixed DimerEraser™ (TaKaRa, Shiga, Japan) on QuantStudio™ 6 Flex qRT-PCR system (Applied Biosystems, Carlsbad, CA, USA). Three biological replicates were analyzed using *β-actin* as an internal standard. qRT-PCR amplification procedure was as described previously [64]. The relative transcript levels of genes were calculated using the 2^−ΔΔCt^ method. All data were analyzed using IBM SPSS Statistics v 25, after ensuring that the data were normally distributed. The independent samples *t*-test was used to identify group differences, which were deemed significant when *p* < 0.05.

## 5. Conclusions

In summary, we conducted a GWAS of growth traits in red tilapia under hyperosmotic stress. Nine SNPs potentially associated with growth traits were detected. Three haplotypes were identified in the QTL region with a significant SNP peak (chr7-47464467) on chr 7; these haplotypes were significantly associated with total body length, body height and body mass. In addition, these haplotypes were all located near *plxnb2*. Combining the results of GWAS and RNA-seq, 11 candidate genes potentially associated with growth were further identified, namely *galnt9*, *esrrg*, *map7*, *mtfr2*, *kcnj8*, *fhit*, *dnm1*, *cald1*, *plxnb2*, *nuak1* and *bpgm*. These candidate genes are involved in the O-glycan biosynthesis signaling pathway and glycolysis/gluconeogenesis, which contribute to growth. These results offer a basis and important resources for further research into the molecular mechanisms of growth traits in red tilapia. Ultimately, this study’s findings will facilitate the development of new tilapia strains with fast growth in saline water.

## Figures and Tables

**Table 1:** Correlations between growth traits of red tilapia.

	Body Mass (g)	Total Body Length (mm)	Body Height (mm)	Caudal Peduncle Height (mm)
Body mass	1			
Total body length	0.897 **	1		
Body height	0.877 **	0.808 **	1	
Caudal peduncle height	0.634 **	0.643 **	0.616 **	1

Note: ** denotes *p* < 0.001.

**Table 2:** The genome significant and suggestive SNPs associated with the growth traits in red tilapia.

Trait	SNP ID	chr	Position	Allele	MAF	PVE (%)	−log(*p*-Value)	Location	Candidate Gene
total body length	chr7-48631309	7	48631309	T/A	0.1558	0.1333	8.38	intergenic	*pnpla8*
	chr15-7606996	15	7606996	A/C	0.1688	0.0306	7.25	intronic	*nbas*
body height	chr2-35563795	2	35563795	T/G	0.3885	0.3873	7.31	intergenic	*tmsb*
	chr7-48631309	7	48631309	T/A	0.1558	0.1333	8.43	intergenic	*pnpla8*
	chr12-11404011	12	11404011	T/C	0.1469	0.0975	7.26	intergenic	*galnt9*
	chr12-11413800	12	11413800	A/C	0.1531	0.1102	7.48	intergenic	*galnt9*
body mass	chr5-37611472	5	37611472	T/C	0.2000	0.0019	7.94	intergenic	*elf3*
	chr7-47464467	7	47464467	T/C	0.4250	0.0036	7.57	intronic	*plxnb2*
	chr7-48631309	7	48631309	T/A	0.1558	0.1333	8.40	intergenic	*pnpla8*
	chr17-19727869	17	19727869	A/C	0.4594	0.0031	7.46	upstream	*acot1*
caudal peduncle height	chr14-37611472	14	37611472	A/G	0.2000	1.3369	7.49	intronic	*lsamp*

## Data Availability

The data and material used in this research are available from the corresponding author on request.

## Supplementary Materials

The following supporting information can be downloaded at: https://www.mdpi.com/article/10.3390/ijms26136492/s1.
