# Supplementary material for: Genome-Wide Association Study and RNA-Seq Analysis Uncover Candidate Genes Controlling Growth Traits in Red Tilapia (Oreochromis spp.) Under Hyperosmotic Stress
Source: Int J Mol Sci. 2025 Jul 5;26(13):6492. doi: 10.3390/ijms26136492 (PMC12249547; doi:10.3390/ijms26136492)
Supplement: Supplementary file 1 [file ijms-26-06492-s001.zip › Table S1.pdf]

Table S1. Descriptive statistics of four growth indicator

Trait (Unit)	N	Min	Max	Mean	SEM	CV(%)
Total body length	80	193	305	238.99	1.64	0.69
body height	80	55	101	78.52	9.97	12.70
Body mass	80	153	528	305.16	6.69	2.19
Caudal peduncle height	80	16	35.9	29.00	0.25	0.86

Table S2. Growth phenotype data of 12 extreme body-size individuals used for transcriptome analysis

Group	Sex	Total body length (mm)	Body height (mm)	Body mass (g)	Caudal peduncle height(mm)
Fast grouping	Male	273.00±7.00^{a}	97.00±1.55^{a}	497.40±15.35^{a}	31.83±1.96^{a}
	Female	269.67±7.31^{a}	88.67±3.38^{b}	469.53±0.26b	32.43±0.72^{a}
Slow grouping	Female	210.00±6.11^{b}	64.33±4.81^{c}	182.63±1.88c	25.17±0.44^{b}
	Male	200.33±3.84^{c}	60.33±2.33^{c}	161.67±6.33d	24.5±0.29^{b}

Table S3. Primers for DEGs in red tilapia

Gene name	Primes (5'to3')
galnt9-F	TATAACAACACCATCACATA
galnt9-R	TTATCATCGTCCTCAGAA
pnpla8-F	AGGAGCAGATTACAGAGA
pnpla8-R	TTGCCACATTAGAGGAAG
bpgm-F	GGCACGGAGAAGTTCAGAGA
bpgm-R	GTTACCAAGCAGGCATTCACA
dnm1-F	CACCAAGGAGAACTGTCTGATG
dnm1-R	ATTCACCACGCCAATGTAACC
plxnb2-F	CACACTGGCACTGGTCTCT
plxnb2-R	ACTCAACACCGCTCTTCACA
nuak1-F	GCACCAATCTGTAAGTCCTGAA
*nuak*1-R	AGTCGGCTCCTCTTCCTCT
cald1-F	ATCAGTAGCAGCAGGCAACA
cald1-R	CCTTCTCCTCAGTCTCTTCCTC
esrrg-F	CTGTCCAGCCACCAATGAGT
esrrg -R	CCTGTCCAGACGAACACCTT
kcnj8-F	TGCCATCACCGTCCTCATC
kcnj8-R	TCTTCCTCACCACCTGTAACC
fhit-F	ACTCTACGCTTCGGACAACA
fhit-R	TCCTGAATGGCGATGGTGAT
actin-F	GTACCACCATGTACCCTGGC
actin-R	TGAAGTTGTTGGGCGTTTGG

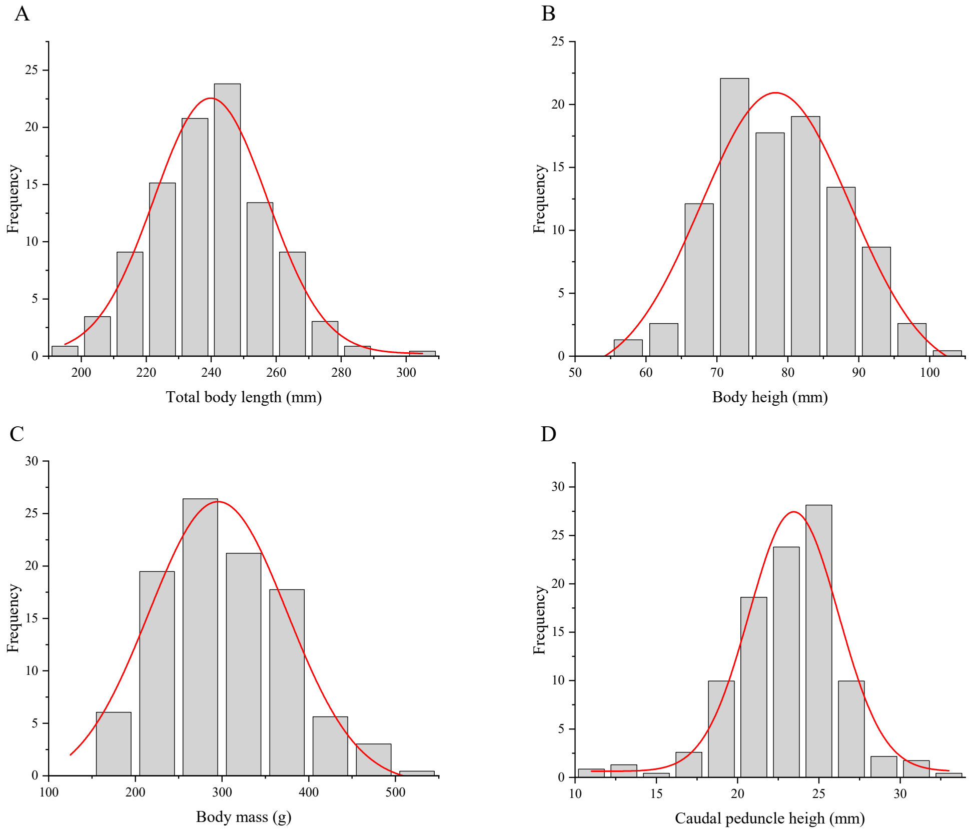

Figure S1. Histogram of frequency distribution of growth traits.

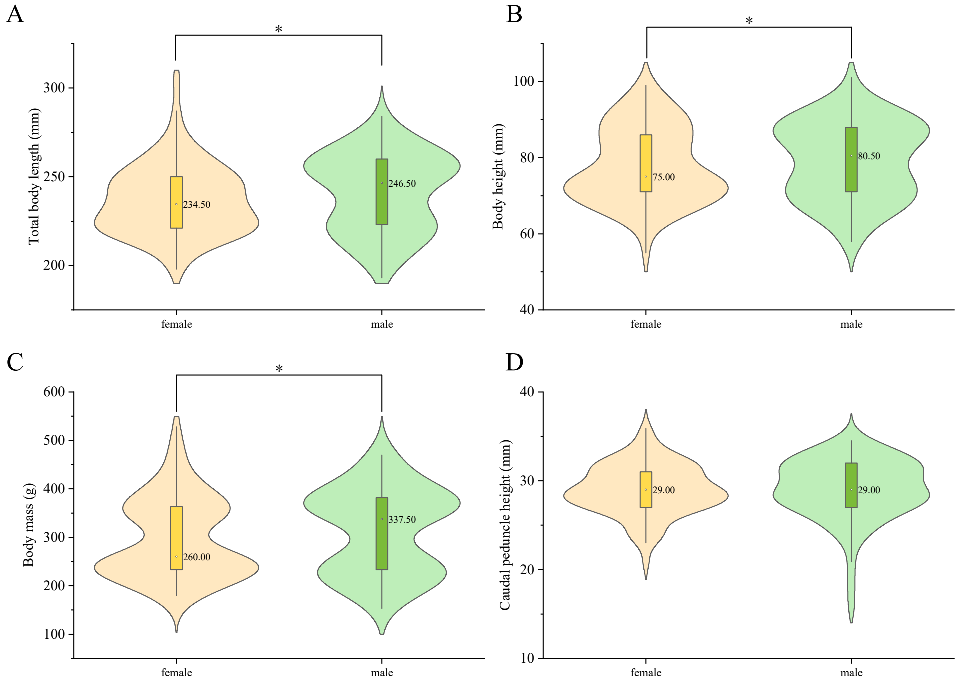

Figure S2. Comparison of growth trait dispersion between male and female individuals

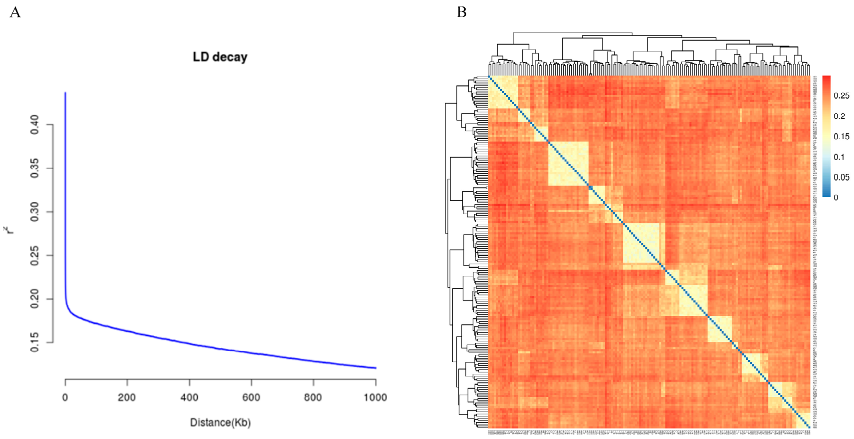

Figuire S3. Analysis of the population structure. (A) Linkage disequilibrium (LD) analysis of 160 red tilapia; Higher r^2 curves indicated that the species had a strong LD. (B) The heatmap of the IBS genetic distance matrix; A square whose color was closer to blue represented a closer relationship; a square whose color was redder presented a more distant relationship.
